# A postural unloading task to assess fast corrective responses in the upper limb following stroke

**DOI:** 10.1186/s12984-019-0483-2

**Published:** 2019-01-28

**Authors:** Catherine R. Lowrey, Teige C. Bourke, Stephen D. Bagg, Sean P. Dukelow, Stephen H. Scott

**Affiliations:** 1 Laboratory of Integrative Motor Behaviour, Centre for Neuroscience Studies, Queen’s University, 18 Stuart St, Kingston, ON K7L 3N6 Canada; 2 Department of Biomedical and Molecular Sciences, Queen’s University, Kingston, ON Canada; 3 Department of Physical Medicine and Rehabilitation, Queen’s University, Kingston, ON Canada; 4 School of Medicine, Queen’s University, Kingston, ON Canada; 5 Hotchkiss Brain Institute, University of Calgary, Calgary, AB Canada; 6 Present Address: Holland Bloorview Kids Rehabilitation Hospital, Toronto, ON Canada

**Keywords:** Proprioception, Stroke, Upper limb, Robotics, Exoskeleton

## Abstract

**Background:**

Robotic technologies to measure human behavior are emerging as a new approach to assess brain function. Recently, we developed a robot-based postural Load Task to assess corrective responses to mechanical disturbances to the arm and found impairments in many participants with stroke compared to a healthy cohort (Bourke et al, J NeuroEngineering Rehabil 12: 7, 2015). However, a striking feature was the large range and skewed distribution of healthy performance. This likely reflects the use of different strategies across the healthy control sample, making it difficult to identify impairments. Here, we developed an intuitive “Unload Task”. We hypothesized this task would reduce healthy performance variability and improve the detection of impairment following stroke.

**Methods:**

Performance on the Load and Unload Task in the KINARM exoskeleton robot was directly compared for healthy control (*n* = 107) and stroke (*n* = 31) participants. The goal was to keep a cursor representing the hand inside a virtual target and return “quickly and accurately” if the robot applied (or removed) an unexpected load to the arm (0.5–1.5 Nm). Several kinematic parameters quantified performance. Impairment was defined as performance outside the 95% of controls (corrected for age, sex and handedness). Task Scores were calculated using standardized parameter scores reflecting overall task performance.

**Results:**

The distribution of healthy control performance was smaller and less skewed for the Unload Task compared to the Load Task. Fewer task outliers (outside 99.9 percentile for controls) were removed from the Unload Task (3.7%) compared to the Load Task (7.4%) when developing normative models of performance. Critically, more participants with stroke failed the Unload Task based on Task Score with their affected arm (68%) compared to the Load Task (23%). More impairments were found at the parameter level for the Unload (median = 52%) compared to Load Task (median = 29%).

**Conclusions:**

The Unload Task provides an improved approach to assess fast corrective responses of the arm. We found that corrective responses are impaired in persons living with stroke, often equally in both arms. Impairments in generating rapid motor corrections may place individuals at greater risk of falls when they move and interact in the environment.

## Background

There has been a dramatic change in diagnostic tools to assess body structure and function over the last few decades. For example, blood tests now provide a range of biomarkers to quantify the function of many organs with measures that are compared to those expected for individuals in good health [17, 60, 61, 68]. In contrast, assessment of brain function has remained relatively unchanged, with the majority of assessments focused on behavior and largely based on the physical or visual inspection of the individual by a clinician using coarse ordinal scales that often have floor and/or ceiling effects.

Robotic technologies offer many advantages as a new approach for neurological assessment as they can provide objective and precise measures of a myriad of behaviours, each designed to assess different aspects of brain function [31, 55]. In recent years, we and others have developed a range of behavioural tasks to quantify sensory, motor and cognitive abilities using an upper limb exoskeleton robotic system. These tasks provide a number of behavioural biomarkers of subject performance and generally have inter-rater reliabilities ranging from good to excellent [6, 7, 16, 26, 29, 37, 57, 62, 65, 66].

An important part of our strategy includes the ability to compare subject performance to a large cohort of healthy subjects, allowing us to objectively identify how performance compares to that expected for neurologically healthy individuals [6, 8, 10, 16, 29, 57, 58, 65]. A key step in this strategy is the generation of normative models of healthy behavior which involves an iterative process to incorporate the effect of factors such as age, sex and handedness. The process also involves data transformation to normal distributions (if required) and the identification and removal of outliers [58]. The development of precise normative models is crucial to ensure that impairments in pathological performance are accurately identified. Ideally, subjects should display similar behavior in a task in order to make it easier to quantify changes from this optimal pattern.

A key aspect of voluntary motor function is the ability to use sensory feedback to generate rapid motor corrections [51, 53]. When holding on to a drink for example, if someone unexpectedly bumps your arm, you must rapidly respond to keep the drink from spilling. In healthy individuals, the motor system can generate complex corrective responses to unexpected disturbances on a very rapid scale, with task-dependent responses emerging as early as 60 ms in muscles after a perturbation [11, 39, 40, 46]. These motor corrections are observed even for very small, imperceptible disturbances that approach the natural variability of movement [12], highlighting that these responses are an integral part of voluntary control and are thought to share similar cortical and sub-cortical pathways to voluntary movement [45, 47], which puts them at risk of impairment following stroke.

We recently developed a Load Task to evaluate the ability of participants to respond to unexpected disturbances of the arm [6]. As compared to healthy controls, many individuals with stroke exhibited a delayed response to the perturbation, took longer to return to the initial position and made larger end-point errors. In some cases, both arms were equally impaired (See also [63]). This bilateral impairment in generating motor corrections may be important clinically as it suggests individuals may have a general impairment in their ability to respond rapidly to unexpected disturbances in the environment, and thus, are at an increased risk of falls.

In order to capture the behavioural impact of impaired rapid motor responses, the Load Task required participants to stabilize their hand within a virtual start target. When the robot bumped their arm, they were asked to return to the target as quickly and accurately as possible [6]. However, subjects commonly co-contract their limb muscles to stabilize against unstable or unpredictable disturbances [18, 24]. In our case, participants could co-contract their upper limb muscles while stabilizing their hand in the start target to oppose the applied load. This strategy limits the amount of limb motion due to muscle properties and short-latency spinal reflexes, termed gain scaling [13, 44]. In order to discourage this co-contraction strategy, subjects were prompted not to respond until they sensed the applied load or “only respond when you feel the load”.

As mentioned above, a key to our approach is the development of normative models of healthy performance in order to provide an objective measure of whether an individual subject is impaired. An important step in this model development is the removal of outliers from the healthy cohort. This process is essential as some healthy subjects may have unidentified impairments in brain function, and thus skew the healthy distribution. In the postural perturbation task, we excluded control subjects that performed outside the 99.9% percentile on any parameter. In our experience with other behavioral tasks, this approach removes ~ 5% or less for all parameters. However, an unusual outcome of the postural perturbation task was that several control subjects responded much more slowly than the majority, leading to a long tail in the distributions for many task parameters. As a result, 13 of the 87 healthy subjects in our cohort were identified as outliers, much higher than 5%. This variation in behavior may reflect that the instruction to relax the arm and “only respond when you feel the load” was interpreted differently across healthy subjects. Some subjects may have responded only after perceiving the stimulus, which takes 100 s of milliseconds, rather than using the fast, automatic motor corrections that begin at 60 ms [53]. Alternatively, it is possible that some subjects could completely relax the arm and maintain the hand at the spatial goal with minimal muscle activity. This means that the motor system was not initially engaged in the postural control task which could result in longer response times to allow the motor system to “re-engage” [53].

Therefore, the purpose of the current study was to develop and validate a new perturbation paradigm. The overall goal of the assessment remained the same, to quantify the ability of participants with stroke to respond quickly and accurately to an unexpected perturbation of the arm. In the new paradigm a background load was applied and then released after an unpredictable time interval. We hypothesized that the new unloading paradigm would decrease variability in control performance compared to the previous loading perturbation, as the motor system must remain engaged during postural control to counter the background load. This background load also naturally discourages co-contraction, which in turn, simplifies the instruction to the subjects.

## Methods

### Participants

Control participants were recruited from the Kingston community and surrounding area. Participants were included in the study if they were older than 18 years of age and could understand task instructions and were excluded if they had any neurologic or musculoskeletal diagnoses affecting the upper limbs. Stroke participants were recruited from the inpatient acute stroke unit and stroke rehabilitation units at Foothills Medical Centre and the inpatient stroke rehabilitation units at Dr. Vernon Fanning Care Centre in Calgary, Alberta and Providence Care Hospital in Kingston, Ontario. Participants with stroke were included in the study if they had a confirmed diagnosis of stroke and could understand the task instructions. Participants were excluded if they had significant medical comorbidities (e.g. angina or active cardiac disease), had a previous stroke, or other neurologic or musculoskeletal diagnoses affecting their upper limbs. All participants provided informed consent prior to participation in the study. This study was approved by the Queen’s University Health Sciences and Affiliated Teaching Hospitals Research Ethics Board (#ANAT042–05), and the University of Calgary’s Conjoint Health Research Ethics Board (#22123).

### Clinical examinations

Clinical evaluations of participants with stroke were administered by a physical or occupational therapist and included the Modified Edinburgh Handedness Inventory for hand dominance as well as the Montreal Cognitive Assessment (MoCA) for assessment of cognitive impairment [42]. The conventional subtests of the Behavioral Inattention Test (BITc) were performed [67]. The BITc consists of 6 pencil and paper tests (e.g. line bisection, letter cancelation, star cancellation, line cancellation, copying, figure drawing) to screen for the presence of visual neglect. The test is scored out of 146 and a value less than 130 is indicative of visual neglect. The Modified Ashworth Scale (MAS) was used to evaluate spasticity at the elbow [5]. The Chedoke-McMaster Assessment of the arm (CMSAa) and hand (CMSAh) was used to assess the upper limb on a 7-point scale reflecting stages of motor recovery following stroke (7–highest recovery stage, 1–lowest recovery) [19]. Strength was manually tested by the therapist and scored on a scale based on the MRC Manual Muscle Testing guidelines (0: (No muscle activity) to 5: (Muscle activation against examiner’s full resistance, full range of motion)) [38]. The Functional Independence Measure (FIM™) was used to rate physical and cognitive disability and level of assistance required, intended to measure the burden of care [20]. The motor portion (FIM motor) measures functional ability, such as washing, dressing, toileting and mobility. The cognitive portion (FIM cognitive) evaluates comprehension, expression, social interaction, problem solving and memory. Participants with stroke were classified by the clinically-determined most affected side of their body and we refer to this side as the “affected side” throughout. Since ~ 30% of individuals with stroke experience impairment in the arm ipsilateral to the lesioned hemisphere [10, 56], we refer to the other side as the “less affected” side.

### Robotic set-up

**Fig. 1:**
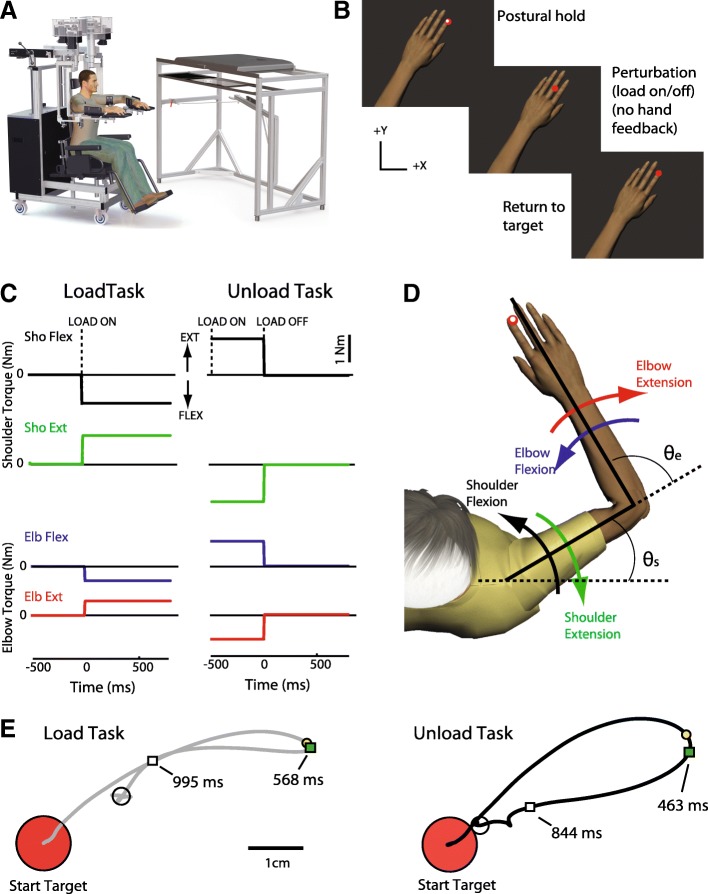
Experimental set-up and task protocol. **a** KINARM exoskeleton robotic system. **b** Task order of events (note hand is included for illustration but hands were occluded from participant view for the entire trial). Postural hold: stabilize white hand feedback marker in red target (with (Unload Task) or without (Load Task) background load). Perturbation: load is turned on (Load Task) or off (Unload Task) and hand feedback marker is removed. Return to Target: return the hand to the target without visual feedback. **c** Load profiles for each direction of perturbation (Sho: shoulder; Elb: elbow; Flex: flexion; Ext: extension). **d** Schematic of the direction of motion following each perturbation type. Representation of how elbow and shoulder angle are defined at the start of the trial (Ɵs is shoulder angle, Ɵe is elbow angle measured at the start position). **e** Hand path after perturbation onset (shoulder flexion trial). The yellow circle indicates the Maximum Displacement of the hand and the green square indicates the Deceleration Time. Return Time (when subject returned within 1 cm of their endpoint location) is indicated by the white square. Endpoint (position at end of trial) is indicated by white circle. Endpoint Error was calculated as the distance between endpoint and the target center

The experimental tasks were performed using the KINARM robotic exoskeleton (BKIN Technologies Ltd., Kingston, Ontario; [52]). The robotic device measures kinematics of the elbow and shoulder and can also apply joint- or hand-based loads. Full detail of the robotic set-up has been previously described [10]. Briefly, participants sat in a modified wheelchair base with the feet resting on adjustable supports. The arms were fully supported in exoskeleton robots with the shoulder and elbow joints aligned with the linkages of the robot arms (Fig. 1a). Plastic arm troughs within the frame were adjusted to support the upper arm and forearm/hand which allowed free arm movement in the horizontal plane. The height of the seat was adjusted for each participant so that the shoulder was abducted ~ 85°. The robot was calibrated for each participant. During testing the arms and hands were occluded from view. A virtual reality system projected visual targets and visual representation of fingertip location on the screen in the same plane as the arm. Position and velocity of the robot were recorded at a sampling rate of 1000 Hz. Subject joint angles and velocities, and hand position, speed, and acceleration were calculated from these values. Hand and joint-based signals were analyzed using MATLAB (Mathworks Inc., Natick, Massachusetts). Signals were filtered using a sixth-order double-pass Butterworth low-pass filter with a cutoff frequency of 10 Hz. All data were collected using the Dexterit-E software program (versions 3.4–3.6, BKIN Technologies Ltd., Kingston, ON, Canada).

### Experimental task

Participants completed two tasks in which perturbations were applied (Fig. 1). These tasks were part of a larger suite of sensorimotor tasks collected using the KINARM device. In one of the perturbation tasks, a mechanical load was applied to the arm (Load Task) displacing it from the start target. In the other perturbation task, participants held against a background load while in the start target and the load was unexpectedly removed (Unload Task).

#### Load task

**Fig. 2:**
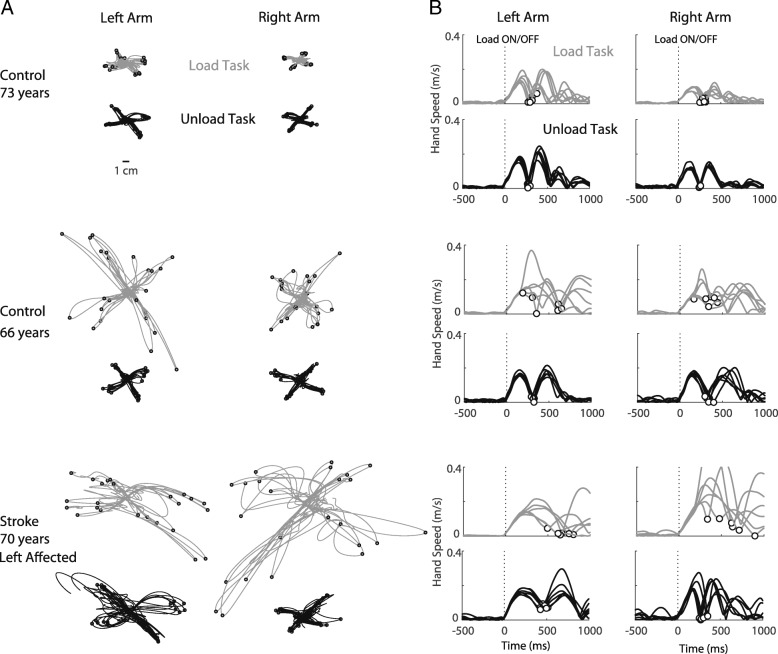
Exemplar participant behaviour. **a** Traces of hand path (left and right arm trials) for each direction of the Load (grey traces) and Unload task (black traces). Hand path is shown for two exemplar control participants (top and middle traces) and an exemplar stroke participant (bottom traces). Small black circles represent the Maximum Displacement calculated for each trial. **b** Hand speed for trials involving shoulder flexion for the left and right arm trials. Dotted lines represent time = 0 ms, when the load was turned on (Load Task) or turned off (Unload Task). Deceleration Time calculated for each trial is represented by the white circles

Participants were instructed to keep the hand (index fingertip) within a virtual start target and to return the hand as quickly as possible to the target any time that the robot ‘bumped’ them out [6]. Each trial began with the participant holding the hand (represented by a white cursor) within the start target. Following a random delay (1750–2250 ms), a flexor or extensor step torque (10 ms rise time) was applied to the elbow (+/− 0.5 Nm) or shoulder (+/− 1 Nm) and remained on for 3 s (Fig. 2). The virtual target was located within the workspace such that the shoulder and elbow angles were 45 ° forward flexion and 75 ° flexion, respectively (Fig. 1d).

#### Unload task

In the Unload Task participants were similarly instructed to maintain the hand within the target and to return the hand to the target as quickly as possible if it was bumped out. In this variant, a background flexor or extensor torque with a rise time of 1000 ms was applied to the elbow (+/− 1 Nm) or shoulder (+/− 1.5 Nm; loads were increased based on pilot testing to provide a similar-feeling perturbation magnitude as the Load Task). Following a random delay (1750–2250 ms) the load dropped to zero over 10 ms. Participants were given 3 s to return to the target after which time the trial timed-out and the next trial began. The virtual target for the Unload Task was moved posteriorly within the workspace (towards the subject) to decrease the likelihood of contacting the far edge of the workspace. The shift brought the shoulder and elbow angles to 30 ° forward flexion and 90 ° flexion, respectively (Fig. 1d).

For each task 8 blocks of 8 trials (2 × 4 load conditions: shoulder flexion, shoulder extension, elbow flexion, elbow extension) were performed for each arm for a total of 128 perturbation trials collected and analyzed for each arm. Trial order was randomized within each block. The arms and hands were occluded from view by a vision blocker. Participants were initially given a practice block of 8 trials with visual feedback of their fingertip position (white 0.5 cm diameter circular cursor). For the remaining trials, the cursor was removed at perturbation onset and only these trials were included in the analyses. All trials were collected on one arm and then the other, and collection order of the arms was randomized. The order of tasks, Load or Unload, was also randomized.

### Task performance measures

Task measures previously developed for the Load Task [6] were used to quantify performance on both tasks in order to compare behavioural performance between the two task variants. In each description ‘perturbation’ refers to either the unexpected loading or unloading of the arm.

#### Posture speed

The 95th percentile of the median hand speed for the 500 ms before perturbation onset. Increased postural speed indicated increased difficulty maintaining the fingertip within the visual target.

#### Deceleration time

Time to reach first minimum hand speed after perturbation onset. This measure quantified how quickly participants were able to slow their arm in response to the perturbation.

#### Return time

Time required for participant to return to within 1 cm of final position at the end of the trial. This indicated how long it took to complete the corrective response within the 3 s time limit. If within a trial the hand was not displaced further than 1 cm, the time required for participants to return to within 0.5 cm of the final position was calculated. It was noted during analysis that in the Load Task, the perturbation load began to ramp down prior to the end of the 3 s window. Therefore, the calculation of Return Time for both tasks was capped at 2250 ms in order to avoid any effects of arm movement when the load ramped down. This only affected subjects with stroke who had extremely long Return Times (i.e. those who did not return to the target) as it may underestimate their actual return times.

#### Endpoint error

Distance from target center at the end of the trial. Endpoint Error quantified the accuracy of the corrective movements. For the reasons outlined above for Return Time, Endpoint Error was calculated at 2250 ms after the perturbation onset.

#### Maximum displacement

Maximum distance the fingertip moved out of the target in response to the perturbation. This parameter measures how effectively participants were able to resist the imposed loading or unloading perturbation.

Joint Velocity Offset (elbow or shoulder) - Difference in time between the first velocity extrema of the directly and indirectly perturbed/loaded joints. For example, when a shoulder flexion perturbation or background load is applied, the shoulder (direct joint) may take a longer or shorter time to respond than the elbow (indirect joint). Joint Velocity Offset was used to quantify multi-joint coordination of the corrective response.

### Statistical analyses

Statistical analyses were performed using Matlab (The MathWorks Inc., Natick, MA, USA). Mean performance of controls and strokes on the two tasks was compared using a signed rank non-parametric comparison to determine differences in mean parameter scores. Kolmogorov-Smirnov tests (K-S tests) were performed to compare the standardized distribution of the control data to a standard normal distribution. The coefficient of variation (CV) was calculated to compare the variability of the performance on each parameter. Each comparison was completed on non-transformed data with the outliers removed.

The performance of participants with stroke on each task was compared to normalized models of behavior calculated from control performance. Details of this process have been outlined previously [29, 58]. Briefly, the control data were normalized using Box-Cox transformations. The control data were fit using multiple linear regression (MLR) to account for age, sex and handedness. Box-Cox equations were then adjusted if necessary to attain a normal distribution. Z-scores were calculated for normal or transformed to normal control parameters. A data point with a Z-score ≤ − 3.29 or ≥ + 3.29 (i.e. 1 in 1000 or outside 99.9% of performance) was classified as an outlier and removed from the control dataset. We refer to these outliers as Parameter Outliers as the data point was removed for a single parameter and the rest of the data for that participant was included for subsequent analysis. The data normalization and outlier removal were repeated for a parameter until no outliers were detected in the dataset.

Task Scores were calculated using all parameter scores to provide a standardized measure of how participants performed overall on the tasks ([58]; See also www.bkintechnologies.com). The parameter Z-scores were transformed such that best performance on a parameter equaled 0 and poor performance was reflected by higher values. The root-mean-square (RMS) of these standardized scores across tasks was performed. The RMS values for all participants were then transformed to normal using Box-Cox equations and further transformed to a Task Score such that 0 equals best performance and poor performance was reflected by higher values. Outliers at this stage were removed if their Task Score was greater than 3.09 which is equivalent to a Z-score of 3.29 (99.9%). We refer to these outliers as Task Outliers as the entire subject was removed from the dataset for the task.

Using the parameter models developed from the control participant data, Z-scores were calculated for parameter scores for participants with stroke. Performance on a given parameter was identified as impaired if it fell outside of 95% of controls; i.e. a Z-score > 1.65 (or for two-tailed parameters Z-score < − 1.96 or > 1.96).

Intra-class correlation coefficients (*r*-values) were used to evaluate the inter-rater reliability of the tasks, comparing task performance of a subset of individuals who were set up in the robot by two different trained KINARM operators.

Correlations between parameter and clinical scores were performed using Spearman’s rank correlation.

## Results

In the current study we compared performance on two robotic tasks designed to assess fast corrective responses in the upper limbs. Both tasks applied an unexpected perturbation to the arm and the goal was to return the hand to the target as quickly and accurately as possible. In the Load Task, a mechanical load was applied in one of four directions whereas in the Unload Task, a background load was applied (in one of four directions) and unexpectedly removed.

### Control participant performance

#### Control: demographic information

We recruited 107 control participants (65 Female/42 Male) who completed both the Load Task and the Unload Task within the same testing session. They performed the task with each arm, for a total of 214 assessments per task. Participant age ranged from 19 to 89 years with a mean age of 41 years (Table 1).

**Table 1:** Participant demographic and clinical information

	Stroke (*N* = 31)	Control (*N* = 107)	
Demographic Info			
Age	60 (26–88)	41 (19–89)	
Sex (M/F)	21 / 10	42 / 65	
Dominant Hand (R/L)	30 / 1	95 / 11	
Stroke Information			
Time since stroke	24 days (2–61)	–	
Affected arm (R/L/B)	15 / 15 / 1	–	
Lesion location	[C SC C + SC Cb Br Cb + Br Mx]^a^	–	
	[6 13 8 1 2 1 0]	–	
Ischemic/hemorrhagic/both	25/ 4 / 2	–	
Clinical test scores			
FIM-motor subscore	75.6 (38–91)	–	
FIM- total scores	104.9 (60–126)	–	
MoCA	24.4 (14–30)	–	
CMSA- arm subscores	[1 2 3 4 5 6 7]	–	
*Affected arm*	[0 1 6 3 5 5 7]	–	
*Unaffected arm*	[0 0 0 0 1 4 19]	–	
Modified Ashworth	[0 1 1+ 2]	–	
*Affected arm*	[23 2 2 0]	–	
*Unaffected arm*	[26 0 0 0]	–	
BITc (/146)	139.5 (112–146)	–	

*C* Cortical, *SC* Subcortical, *C + SC* Cortical + Subcortical, *Cb* Cerebellum, *Br* Brainstem, *Cb + Br* Cerebellum + Brainstem, *Mx* Mixed

#### Control: exemplar participant behaviour

Performance of two control participants is illustrated in the top two panels of Fig. 2 (top and middle traces) and represents the two patterns of behavior that we typically observed. The top traces illustrate a 73 year old participant with similar behavior on both the Load and Unload task. The participant’s arm was minimally displaced by loading or unloading, and displayed a relatively consistent return profile (hand path, top traces panel A and hand speed, top traces panel B). The responses to the Load Task for this participant do show more variability, however, than the Unload Task as evidenced by more variable Deceleration Time (white circles on hand speed traces). The middle traces illustrate a 66 year old participant with more variable performance on the Load Task compared to the Unload Task. The hand displacements are much larger and more variable in the Load Task and hand speed traces and Deceleration Time were also more variable than for the Unload Task.

#### Control: group performance

**Fig. 3:**
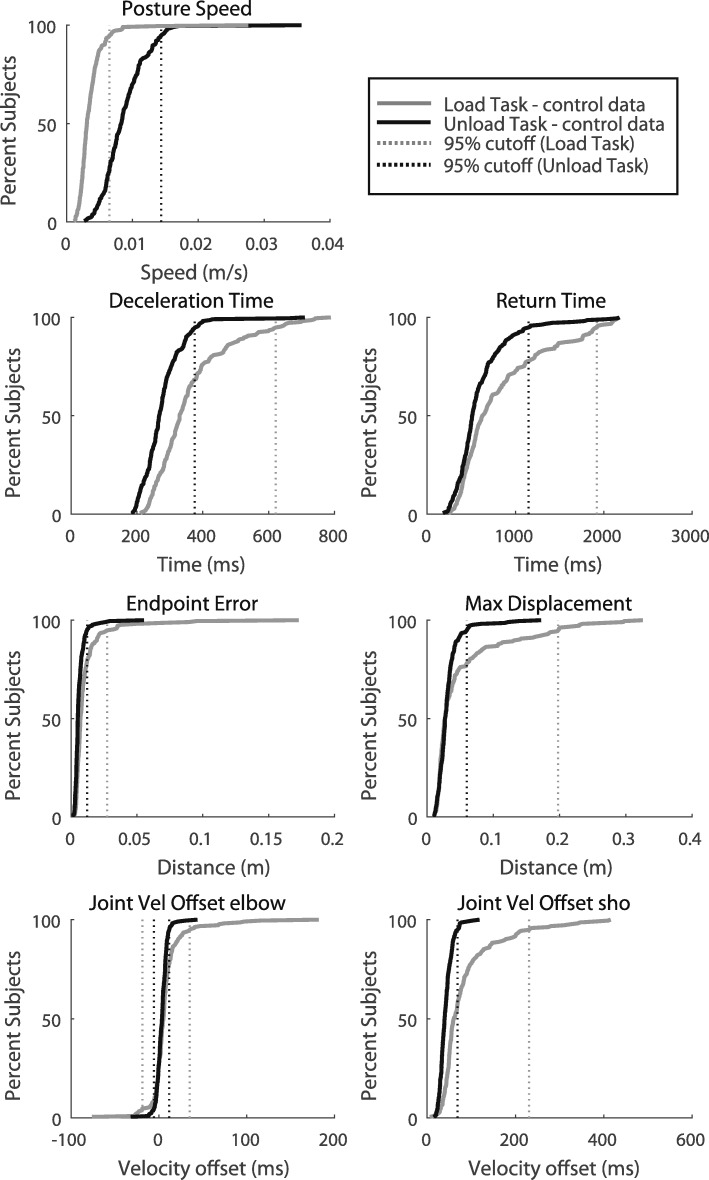
Cumulative sums of each parameter reflecting the distribution of control performance on each task. Control parameter performance is displayed for both the Unload (black curves) and the Load Task (grey curves). Dotted lines represent the 95th percentile of control participant behavior for both the Load (grey lines) and the Unload (black lines) task. Note that Joint Velocity Offset elbow is a two-sided distribution so lines representing the 2.5 and 97.5 percentiles are shown

Figure 3 highlights that even though the Load and Unload Tasks are very similar, there can be clear differences in performance by healthy control subjects. The parameter means were significantly decreased by ~ 25–50% for the Unload Task (Fig. 3, Table 2). The exception is Posture Speed which was significantly increased in the Unload Task (by ~ 40%). For most parameters, the distribution range for the Unload Task was narrower and less skewed (shorter rightward tails). Correspondingly, the Coefficient of Variation (CV) was always smaller for the Unload Task compared to the Load Task (Table 2). Thus, it is unsurprising that more parameters in the Unload Task were initially normally distributed. Prior to normalization via Box-Cox transforms, the baseline normality of the distributions was tested using K-S tests. Four of the 7 Unload parameters were normally-distributed (H = 0, *p* < 0.05; Table 2). In contrast, none of Load parameters were normally distributed (H = 1, *p* > 0.05). The 3 Unload parameters that were not normally distributed (Return Time, Max Displacement and Endpoint Error) had a lower K-S test statistic for the Unload Task compared to the Load Task indicating less distance from the expected normal distribution curve.

**Table 2:** Control participant task performance

	Mean		CV (%)		K-S test (H, ks-stat)		Outliers removed	
	Load	Unload	Load	Unload	Load	Unload	Load	Unload
Posture Speed (cm/s)	0.35^**^	0.86	46.1	34.2	1, 0.12	0, 0.07	1	1
Deceleration Time (ms)	371.7^**^	275.2	31.8	18.7	1, 0.16	0, 0.08	0	2
Return Time (ms)	881.6^**^	619	56.6	49.1	1, 0.18	1, 0.15	0	0
Max Displacement (cm)	5.5^**^	3.1	111.7	39.3	1, 0.24	1, 0.17	0	3
Endpoint Error (cm)	1.2^**^	0.6	159.9	45.7	1, 0.28	1, 0.1	0	4
JVO elbow (ms)	5.2^*^	3.29	218.1	171.7	1, 0.11	0, 0.04	17	4
JVO shoulder (ms)	82.7^**^	41.8	71.5	37.5	1, 0.21	0, 0.08	3	1
TASK outliers (participants completely removed based on Task Score)							8 (7.4%)	4 (3.7%)

^*^p < 0.05; ^**^ *p* < 0.001

Following the normalization and outlier removal algorithms, all parameters were able to be transformed to a normal distribution. As a part of the normalization process, fewer outliers were removed for the Unload Task compared to the Load Task (Table 2). Overall, only 4 Task Outliers were removed for the Unload Task, whereas 8 Task Outliers were removed from the Load Task. At the parameter level for the Unload Task, 4 or fewer data points were removed as parameter outliers (from the total 214 data points for each parameter, i.e. 1.8% or fewer data points removed for each parameter). Only Return Time had no parameter outliers removed. In the Load Task, a large range of outliers was noted across parameters. Four parameters (Deceleration Time, Return Time, Max Displacement and Endpoint Error) had no parameter outliers removed, while Joint Velocity Offset (elbow) had 17 parameter outliers removed (7.9% of the data for that parameter). Joint Velocity Offset (shoulder) had 3 parameter outliers, and Posture Speed had 1.

### Stroke participant performance

#### Stroke: subject demographic information

Initially 36 participants with stroke were recruited to participate in the current study but 5 participants were unable to complete either the Unload or Load Tasks with their affected arm and were not included in the analyses (See Discussion). Therefore, 31 subjects with subacute stroke completed both the Unload and Load Tasks; demographic and clinical information is provided in Table 1. The date of the robotic testing session ranged from 2 to 61 days post-stroke across participants. There was an approximately equal amount of participants with stroke that were right and left affected (14 RA, 17 LA). Most participants had an ischemic stroke (25), 4 participants had a hemorrhagic stroke and 2 had ischemic strokes with hemorrhagic transformation. Our group of subjects with stroke displayed a broad range of FIM scores (60–126), but a median score of 109 indicated many subjects had mild disability (108 for LA subjects and 107 for RA subjects). CMSA arm scores ranged from 2 to 7 for the affected arm and 5–7 for the less affected arm. BITc scores ranged from 112 to 146 with a mean of 139.5. Only two individuals had BITc scores less than 130, indicative of neglect.

#### Stroke: exemplar participant behaviour

The performance of a 70 year old participant with stroke is illustrated in the bottom traces of Fig. 2. The performance on both tasks is highly variable with larger hand displacements on each task. Similar to controls, performance of the participant with stroke is more variable for the Load Task compared to performance on the Unload Task.

#### Stroke: group performance

**Fig. 4:**
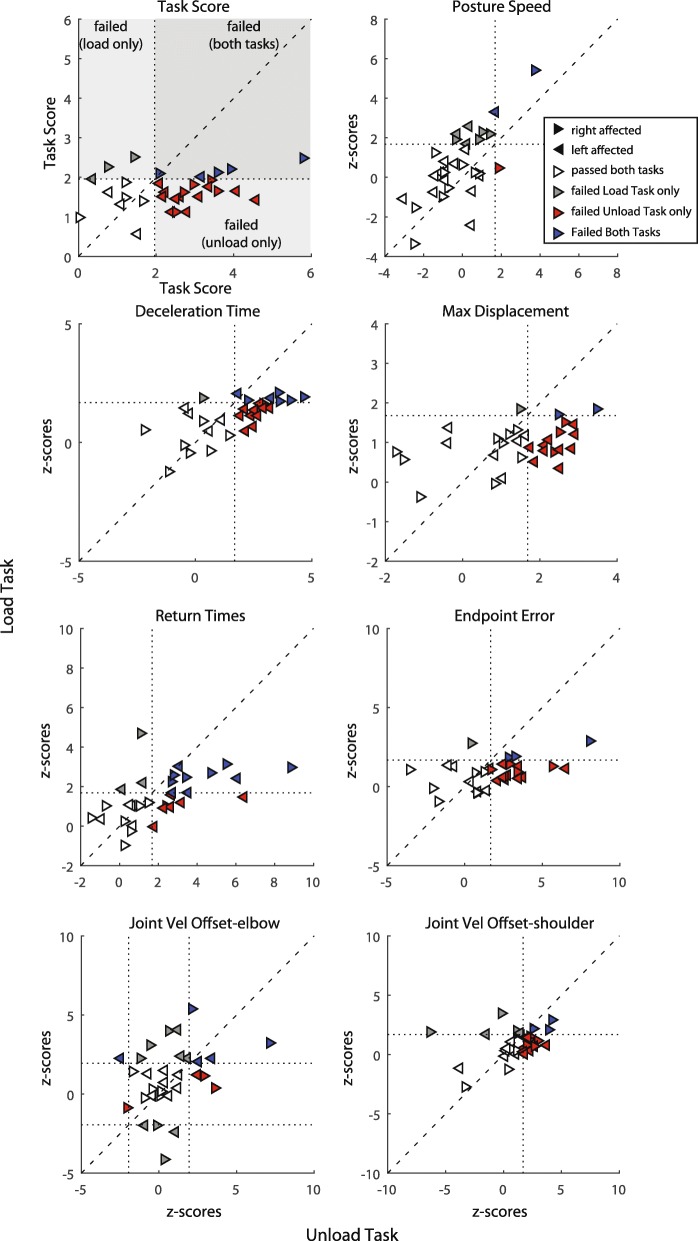
Standardized stroke performance on the tasks with the affected arm. Overall task score and parameter Z-scores are presented. Dotted lines represent the cutoff values (1.96 for task score and 1.65 for Z-scores). The comparison for Joint Velocity Offset (elbow loads) is two-tailed so Z-score cut off values were − 1.96 and + 1.96. Triangles reflect individual stroke participants: right-facing is right affected and left-facing are left affected participants. Based on whether the participant exceeded the cut off values on each task they are colour-coded in 4 quadrants: failed both tasks (blue), failed Unload Task only (red), failed Load Task only (grey), passed both tasks (white)

Individual standardized stroke performance on each parameter is illustrated in Fig. 4. Overall, more subjects were identified as impaired on the Unload Task than the Load Task. Based on Task Score for the affected arm, 68% of participants failed the Unload Task (Task Score > 1.96) compared to 23% who failed the Load Task (Table 3, Fig. 4). However, similar failure rates between tasks were found for the less affected arm Task Scores, 23% failed the Unload Task with their less affected arm and 26% failed the Load Task.

**Table 3:** Stroke participant task performance

	Parameter Scores (mean)				Failure rate (%)				Z-score range				Reliability	
	Aff Arm		Less Aff Arm		Aff Arm		Less Aff Arm		Load		Unload		ICC (*r*-values)	
Parameters	Load	Unload	Load	Unload	Load	Unload	Load	Unload	Min	Max	Min	Max	Load	Unload
Posture Speed (cm/s)	0.52	0.95	0.62	1.09	29	10	26	6	−3.35	5.44	−3.04	3.7	0.74^*^	0.76^*^
Deceleration Time (ms)	516	379	430	289	26	61	10	13	−1.22	2.12	−2.18	4.64	0.8^*^	0.86^*^
Return Time (ms)	1511	1219	1402	970	10	52	6	29	−0.38	1.86	−1.72	3.46	0.9^*^	0.89^*^
Max Displacement (cm)	10.8	7.3	10.7	6	13	55	13	16	−0.93	2.88	−3.55	8.08	0.97^*^	0.91^*^
Endpoint Error (cm)	5.2	3.6	4.9	2.8	42	55	26	29	0.98	4.7	−1.44	8.81	0.9^*^	0.91^*^
JVO elbow (ms)	21	7.4	28	6	45	29	35	10	−4.14	5.39	−2.48	7.16	0.78^*^	0.47^*^
JVO shoulder (ms)	142	62	131	48	29	48	23	16	−2.71	3.43	−6.31	4.31	0.76^*^	0.55^*^
Task Score	–	–	–	–	23	68	26	23	0.57	2.51	0.01	5.8	0.7^*^	0.72^*^

^*^p < 0.05

Failure on a parameter was determined as a Z-score > 1.65 (or − 1.96 > Z-score > + 1.96 for two-tailed parameter such as Joint Velocity Offset elbow). The failure rate for the affected arm of participants with stroke was greater for the Unload Task for all parameters (with the exception of Posture Speed and Joint Velocity Offset elbow (Table 3)). The Unload Task parameter that captured the largest amount of impairments was Deceleration Time, where 61% of participants failed (compared to 26% in the Load Task). For the less affected arm, failure rate was higher on the Unload Task for 4 of the 7 parameters (Deceleration Time, Return Time, Endpoint Error, Max Displacement) whereas fewer participants failed Posture Speed and Joint Velocity Offset elbow and shoulder compared to the Load Task. Of note, the Unload Task generally had a larger range of Task Scores (0.01–5.80) than the Load Task (0.57–2.51; Table 3, Fig. 4). Similarly, each Unload Task parameter (with the exception of Posture Speed) had a larger range of Z-scores than the Load Task parameters.

**Fig. 5:**
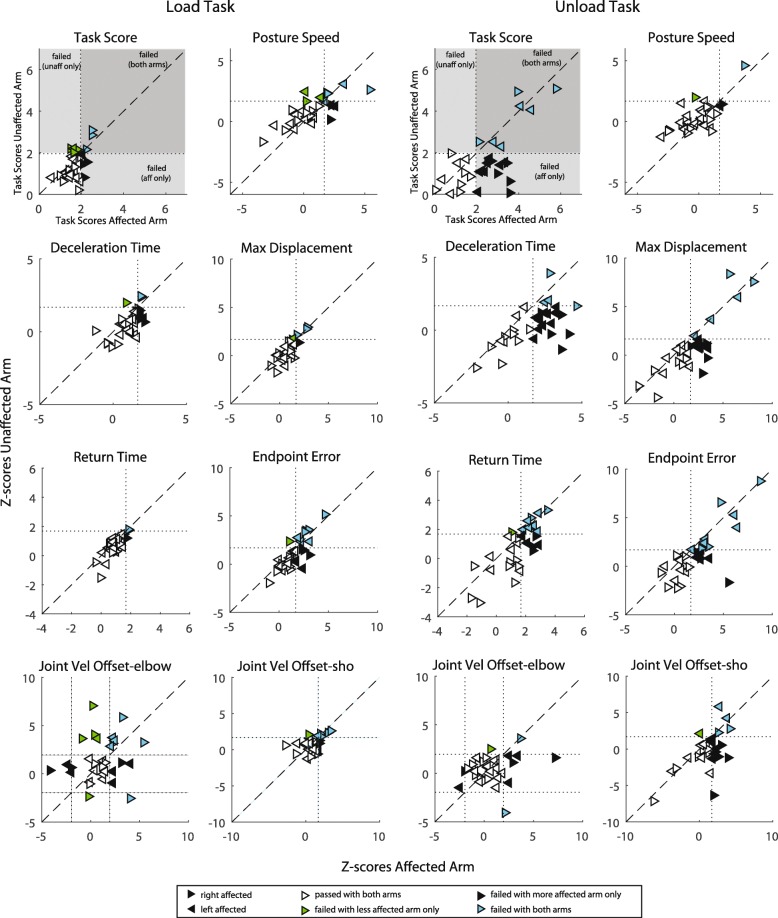
Task Scores and Z-scores plotted for the affected and less affected arms for each task. Dotted lines represent the cutoff for passing the task or parameter. Triangles reflect individual stroke participants: right-facing are right affected and left-facing are left affected participants. Based on whether the participant exceeded the cut off values on each arm they are colour-coded in 4 quadrants: failed task with both arms (cyan), failed task with affected arm only (black), failed task with less affected arm only (green), passed both task with both arms (white)

For the Unload Task, 23% of participants failed the task with both arms and all participants who failed with their less affected arms also failed with their affected arm (Fig. 5). Only 10% of participants failed the Load Task with both arms. Surprisingly, 16% of participants actually failed the Load Task with their less affected arm only and passed the task with their affected arm.

#### Inter-rater reliability

Inter-rater reliability of the parameters was evaluated using an intraclass correlation coefficient (ICC, Table 3). Inter-rater data were collected for 67 participants for the Unload Task (*N* = 11 stroke participants). Values for the Unload Task ranged from 0.47 to 0.93. Joint Velocity Offset (both elbow and shoulder loads) had the lowest ICC scores (0.47 and 0.55) indicating fair reliability. The ICC for Posture Speed was 0.76 reflecting a good inter-rater reliability. All other parameters ICC scores were above 0.8 reflecting excellent inter-rater reliability. Inter-rater data were collected for 18 participants on the Load Task (N = 11 stroke participants). The ICC values for the Load Task ranged from 0.74 to 0.97 reflecting good to excellent inter-rater reliability.

#### Correlation with clinical scores

Performance on the Unload Task was significantly correlated with more standard clinical scores than performance on the Load Task (Table 4). Similar to our previous findings [6], performance on the Load Task with the affected arm did not significantly correlate with standard clinical test scores, with the exception of Deceleration Time and Posture Speed. Affected arm Task Scores for the Unload Task were negatively correlated with CMSA scores (arm + hand) indicating that poor performance on the task was associated with poor scores for the arm and hand. The CMSA score was also negatively correlated with Deceleration Time, Return Time, Max Displacement, and Joint Velocity Offset. Scores of the FIM were negatively correlated with Deceleration Time and Max Displacement. Interestingly, affected arm Posture Speed for both the Load and Unload Tasks was positively correlated with CMSA (arm and hand) indicating that lower posture speed was associated with poor CMSA scores.

**Table 4:** Correlations with clinical scores

Parameter	FIM (*r*-value)		CMSA (*r*-value)		Strength (*r*-value)	
	Load	Unload	Load	Unload	Load	Unload
Posture Speed (cm/s)	0.12	0.27	0.39	**0.56** ^*^	0.31	0.42^*^
Deceleration Time (ms)	**−**0.41^*^	−0.38^*^	**−0.48** ^*^	**−0.52** ^*^	− 0.35	−0.32
Return Time (ms)	−0.03	−0.33	0.08	**−**0.41^*^	0.03	−0.28
Max Displacement (cm)	−0.1	−0.37	− 0.13	**−**0.39^*^	− 0.05	−0.17
Endpoint Error (cm)	−0.21	−0.25	− 0.15	−0.3	− 0.13	−0.23
JVO elbow (ms)	−0.14	−0.2	− 0.14	−0.25	− 0.21	−0.31
JVO shoulder (ms)	0.02	−0.26	−0.05	− 0.41^*^	−0.05	− 0.09
TASK SCORE	0.07	−0.35	0.17	**−**0.41^*^	0.03	0.01

^*^ *p* < 0.05, bolded and ^*^ *p* < 0.0016 (*p* value corrected for multiple comparisons)

## Discussion

### Motor performance in the Unload Task is more consistent across healthy subjects than the Load Task

While both the Load and Unload Tasks quantified how subjects respond to an unexpected disturbance, there are clear differences in the performance of neurologically healthy subjects between these two tasks. For most task parameters, the distribution of performance was broader and tended to be more skewed towards larger values for the Load compared to the Unload Task. Thus, performance at the 95th percentile for healthy subjects was generally worse for the Load Task. The only exception was Posture Speed which displayed larger values for the Unload Task most likely due to the initial background load. The algorithms also found fewer outliers for the Unload Task when data for each parameter were transformed to normal distributions.

It is interesting to speculate as to the reason why there were such large differences in subject performance across these seemingly related tasks. It is possible that these differences in performance are related to differences in feedback processing when loading versus unloading the arm. However, previous work has shown that muscle responses to perturbations that unload the arm share similar properties to those that load the arm. Loading and unloading responses both generate small, but similar-timed short-latency spinal responses that appropriately excite or inhibit muscle activity, respectively [2, 3, 43]. As well, unexpected unloading of pre-excited, shortened muscles results in task-dependent muscle inhibition as early as 60 ms [41], similar in timing to task-dependent increases observed for unexpected loading. These rapid muscle responses involve transcortical feedback pathways, share many attributes with voluntary control [54] and contribute to the rapid goal-directed corrective movements in the current tasks (i.e. deceleration of the hand within ~ 500 ms). Thus, rapid feedback processing appears to be similar whether the limb is loaded or unloaded.

A more likely explanation is that different subjects may have used different strategies for the Load Task due to the instructional prompts to “relax” the arm while waiting in the start target and “respond when you feel the perturbation”. These prompts were necessary to discourage co-contraction in some instances but may have inadvertently introduced a decisional component to the task. In particular, some subjects may interpret this instruction to mean that they should respond only after perceiving the load rather than simply engaging the fast, context-dependent feedback pathway. The rightward skew seen for several parameters in the Load Task (Response Time, Deceleration Time, Max Displacement) is similar to the ex-Gaussian distribution often observed for reaction time tasks [4, 22, 48]. The rightward skew can reflect the existence of two underlying strategies [30], and specifically the existence of a decisional component [28]. The exponential component of the ex-Gaussian distribution of reaction times (i.e. the long rightward tail) is thought to represent the decisional component of the response. While this has primarily been investigated at the individual subject level, the presence of a portion of healthy subjects with much longer response times and displacements in our distribution suggests that these subjects predominantly used a strategy to respond only after perceiving the load, whereas most subjects respond quickly and presumably relied primarily on fast automatic feedback pathways to counter the applied load.

Another possible contributing factor to the broader distribution of task parameters for the Load Task may be related to whether or not subjects were actively engaged in a postural control task at the beginning of each trial. Specifically, some subjects in the Load Task may have been able to completely relax their arm and still maintain their hand at the start target. In these subjects, it may take additional time for the system to re-engage in a motor task when the arm was perturbed [53]. In contrast, the background load for the Unload Task required the subject to be actively engaged to maintain postural control. Thus, the background load, which was originally employed to discourage co-contraction, may have an inherent additional advantage in that it provides consistent engagement of the motor system reducing inter-subject variability in healthy controls.

### The Unload Task identified more impairments in subjects with stroke than the Load Task

In general, the Unload Task identified more impairments in participants with stroke. Sixty-eight percent of participants failed the Unload Task with their affected arm (Task Score > 1.96) compared to 23% who failed the Load Task. The failure rate at the parameter level was higher for the Unload Task for the majority of parameters. Further, a broader range of Task Scores and Z-scores were found for the Unload Task compared to the Load Task. A broad range of scores allows for higher resolution in scoring participant performance and suggests that the Unload Task may be better suited to distinguish between levels of impairment, for example mild, moderate and severe. Deceleration Time for the Unload Task identified the largest number of impairments with 61% of participants with stroke failing this parameter, indicating that the majority of participants took longer to slow their arm after the load was removed. In contrast, only 26% of participants with stroke failed this parameter in the Load Task. We expect that reductions in long-latency and early voluntary muscle responses seen following stroke [15] likely contribute to the delays in deceleration time observed for participants with stroke in our tasks. Previous work has shown less inhibition of muscle activity following unloading in participants with stroke [35] which may impact the ability to slow the arm when the load is removed in the current task.

The fact that the Unload Task identified many more subjects with stroke as impaired is likely due to the smaller range of performance observed for healthy controls compared to the Load Task. We observed a similar effect with a task we developed to quantify bimanual motor function [29]. The task was designed with multiple levels that increased in difficulty, with the assumption that more participants with stroke would be identified as impaired as the task difficulty increased. However, impairment rate dropped as the levels became more challenging, primarily because the behavior of the control population became more variable, making it more difficult to identify impairments in the behaviour of participants with stroke. In fact, the first (and simplest) level of the task was the most effective at identifying impairments in behavior because of the narrow range of control behaviour. This demonstrates that two key design features for behavioural tasks to quantify impairments in brain function are simple task goals and motor actions that minimize the range of performance for a healthy control population.

There was a substantive drop in the number of subjects with stroke identified as impaired between the present study and our previous study (Bourke et al., 2014). This is likely due to changes in the procedures for developing normative models and outlier removal between studies. In the previous study, if a control subject’s performance was outside the 99.9th percentile for a single parameter, the subject’s data were excluded for all parameters. This led to 15% of subjects being removed from the dataset. In contrast, in the current study, performance beyond the 99.9th percentile for a single parameter led to the subject’s data point only being removed for that parameter. Complete removal of a subject’s data only occurred if their Task Score was beyond the 99.9th percentile. This led to fewer outliers being completely removed in the present study (3.7% for Unload Task and 7.4% for Load Task). The larger number of subjects removed from the dataset in the previous analyses likely lowered the 95% cutoff values substantially, resulting in more individuals with stroke identified as impaired. However, the number of identified impairments in that analyses was likely too high and captured a number of false positives. Interestingly, the present Unload Task identified about the same number of subjects as impaired as in the previous study, but with far fewer outliers removed from the control dataset. Thus, the present Unload Task likely provides a more accurate measure of impairment related to a subject’s ability to respond to a mechanical disturbance.

It is also interesting to note that impairments found with the Unload Task were related more strongly to standard clinical measure of upper limb recovery and general functional ability compared to the Load Task. Unload Task Scores were significantly correlated to CMSA scores of the arm and hand but Load Task Scores were not. Further, more individual parameters were correlated with CMSA scores for the Unload Task. This suggests that impairments found with the Unload Task are more reflective of upper limb recovery. Few parameters were correlated to the FIM, which is not surprising given that the FIM covers so many domains of behavior (i.e. gait, posture, toileting, grooming etc). However, it is interesting to note that Deceleration Time was correlated with both the FIM (and the CMSA) for both the Unload and Load Tasks, suggesting that the ability to stop the arm following a perturbation may be related to both upper limb recovery and general functional ability.

One drawback of the Unload Task is that subjects must be able to counter a small load when maintaining their hand at a central target. Although the background load is relatively small, subjects with severe weakness may not be able to counter the background load to maintain their hand at the spatial target. In the current sample, 5 participants of the initial 36 participants with stroke recruited for this study were unable to complete the Unload Task with their affected arm. However, 4 of the 5 participants were also unable to stabilize in the start target for the Load Task suggesting that both tasks require a minimal amount of strength and/or coordination to maintain the hand at the spatial goal. These individuals were severely impaired with paresis of their affected arm and CMSA arm scores ranging from 1 to 3.

### Bilateral impairments

Approximately one-third of participants who failed the Unload Task with their affected arm also failed with their clinically-defined ‘less affected arm’. In some cases, following a unilateral stroke, impairments can observed in both arms although the contralesional limb is always more impaired than the ipsilesional arm [10, 14, 36, 56, 59, 69]. Ipsilesional impairments may be due to direct damage to the corticospinal fibers that project to the ipsilateral side [23] or from the influence of the lesioned hemisphere on the intact hemisphere [1]. What is unusual in the present study is that some subjects are equally impaired in generating feedback corrections in both arms, as demonstrated by similar Z-scores for both arms. Previous studies have shown that the long-latency muscle response can be diminished in both arms in some individuals following stroke [34]. As well, reflex coordination [64] and task-dependent modulation [63] of the long-latency response are impacted in both arms following stroke, all of which may contribute to similar levels of impairments in corrective movements for both arms.

Another possibility is that equal impairments in both limbs may be due the hemispheric lateralization of specific aspects of motor tasks [21, 25, 49, 50] where damage to one hemisphere may impact the motor ability of both arms. However, this bilateral impairment was observed in subjects with stroke in either hemisphere. Alternatively, both hemispheres may be equally involved in certain aspects of control. Previous work has shown that neural activity in dorsal premotor cortex is tuned to movements of either arm [9]. Thus, the rapid engagement of motor corrections may involve contributions of both contra- and ipsilateral hemispheres.

### Clinical implications

The Unload Task shows that individuals with stroke can have significant impairments in rapid corrective responses following mechanical disturbances to the arm. Evidence is mounting that the neural pathways associated with rapid feedback responses are closely linked with voluntary control [54]. Impairments in this task may signal global problems in voluntary control of the arm as well as the inability to respond appropriately to unexpected disturbances when interacting in the environment, which may lead to increased risk of falling if the disturbance is large enough to challenge postural stability.

Although it is important to assess the ability of individuals to rapidly respond to disturbances, very few commonly used clinical scales for the upper limbs include mechanical disturbances. This is most likely because of the difficulty of providing a consistent perturbation between evaluators and/or across sessions, highlighting the advantage of robotic technology. If an impairment is identified, it would be advantageous to include mechanical disturbances into an upper limb rehabilitation program, either through the use of a robotic therapy device or by the therapist, a practice that is already being used for whole-body posture rehabilitation programs [27, 32, 33].

The proposed Unload Task is limited to the shoulder and elbow joints, as the robotic device only measures and applies loads at these two joints. However, the bilateral impairments that were commonly observed suggest this task may identify global rather than just joint-specific impairments. Future work should investigate if results at the shoulder and elbow can be used as a proxy for other joints or body segments. Another limitation is that robotic devices for assessment are currently not widely available for clinical use. Work is ongoing to validate this and other assessment tasks and to make these and the robotic technologies more broadly available.

## Conclusion

The Unload Task provides an improved tool for the assessment of corrective responses. Unloading the arm is associated with more straightforward instructions which results in a more consistent response from control participants with fewer outliers removed. The Unload Task identifies more impairments in behavior in participants with stroke which are in turn, more strongly related to standard clinical measures of arm recovery. Taken together these results highlight the benefits of the Unload Task as an assessment tool for evaluating rapid corrective responses following mechanical disturbances to the arm.

## Abbreviations

BITc: Behavioural Inattention Test (conventional portion)
CMSA: Chedoke-McMaster Assessment
CV: Coefficient of variation
FIM: Functional Independence Measure
ICC: Intraclass correlation
K-S test: Kolmogorov-Smirnov test
LA: Left affected
MAS: The Modified Ashworth Scale
MoCA: Montreal Cognitive Assessment
RA: Right affected
RMS: Root-mean-square
